# Physiological Signal Analysis for Evaluating Flow during Playing of Computer Games of Varying Difficulty

**DOI:** 10.3389/fpsyg.2017.01121

**Published:** 2017-07-04

**Authors:** Yu Tian, Yulong Bian, Piguo Han, Peng Wang, Fengqiang Gao, Yingmin Chen

**Affiliations:** ^1^Department of Psychology, Shandong Normal UniversityJinan, China; ^2^Department of Computer Science and Technology, Shandong UniversityJinan, China

**Keywords:** flow experience, shyness, physiological signal, person–artifact–task model, computer game

## Abstract

Flow is the experience of effortless attention, reduced self-consciousness, and a deep sense of control that typically occurs during the optimal performance of challenging tasks. On the basis of the person–artifact–task model, we selected computer games (tasks) with varying levels of difficulty (difficult, medium, and easy) and shyness (personality) as flow precursors to study the physiological activity of users in a flow state. Cardiac and respiratory activity and mean changes in skin conductance (SC) were measured continuously while the participants (*n* = 40) played the games. Moreover, the associations between self-reported psychological flow and physiological measures were investigated through a series of repeated-measures analyses. The results showed that the flow experience is related to a faster respiratory rate, deeper respiration, moderate heart rate (HR), moderate HR variability, and moderate SC. The main effect of shyness was non-significant, whereas the interaction of shyness and difficulty influenced the flow experience. These findings are discussed in relation to current models of arousal and valence. The results indicate that the flow state is a state of moderate mental effort that arises through the increased parasympathetic modulation of sympathetic activity.

## Introduction

Since Csikszentmihalyi’s systematic description of flow in his 1975 work *Beyond Boredom and Anxiety*, this optimal experience has become a crucial concept in related research. He noted that artists become entirely immersed in their projects, working feverishly to complete them, and then lose all interest in their work after completion. In more recent research, this optimal experience has been investigated in various domains such as computer game playing (Rheinberg et al., 2003; Keller and Bless, 2008; Moller et al., 2010; Keller et al., 2011; Bressler and Bodzin, 2013; Peifer et al., 2014; Harmat et al., 2015), musical instrument performance (De Manzano et al., 2010), course learning (Engeser and Rheinberg, 2008; Schaik et al., 2011), mountain hiking (Wöran and Arnberger, 2012), and team performance (Aubé et al., 2014). In addition, studies have suggested that flow has positive effects on psychological well-being (Clarke and Haworth, 1994; Asakawa, 2004, 2009) and quality of life (Csikszentmihalyi, 1990). These observations motivated a scientific investigation of a flow assessment system.

### Mediation of Flow Precursors

**FIGURE 1:**
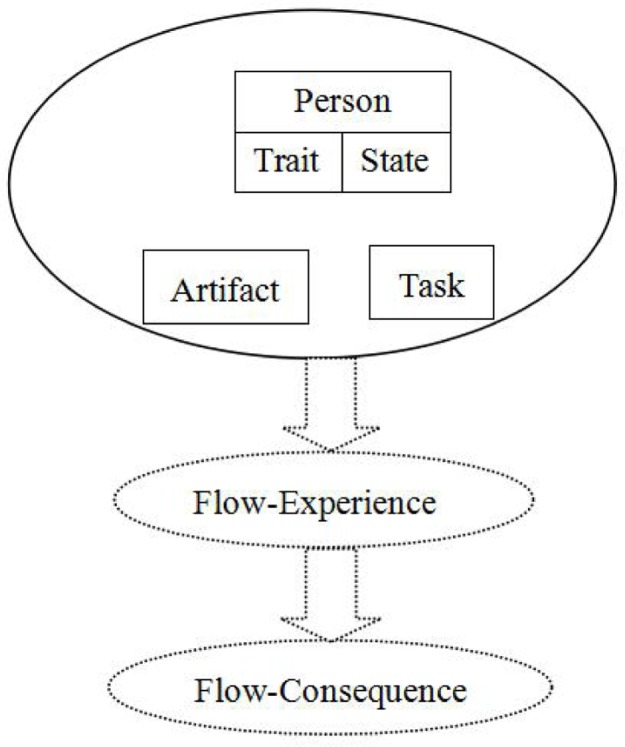
Person–task–artifact model (Finneran and Zhang, 2003).

Previous studies have suggested that computer-mediated environments (CMEs) are an appropriate context for assessing flow experience. Such environments are conducive to flow because they facilitate flow-precursor interactions through clear artifact goals, immediate feedback, and task characteristics (Finneran and Zhang, 2003; Mauri et al., 2011). To clarify the causes of the flow experience in CMEs, Finneran and Zhang (2003) developed the person–artifact–task (PAT) model (**Figure 1**) to evaluate constructs and ambiguous flow-related operationalizations. This model uses the following three stages of flow as a framework: flow precursors, flow experience, and flow consequences. The precursors of flow are person (P), artifact (A), and task (T). The flow experience itself is composed of concentration, loss of self-consciousness, time distortion, and telepresence. The consequences of flow are positive affect and an autotelic experience. The PAT model focuses on flow precursors, and the model structure suggests that flow experience is the consequence of interactions among these precursors. To psychometrically assess the viability of this model, Schaik et al. (2011) measured flow experience in an immersive virtual environment for collaborative learning. The results indicated that flow experience is mediated by its precursors.

**FIGURE 2:**
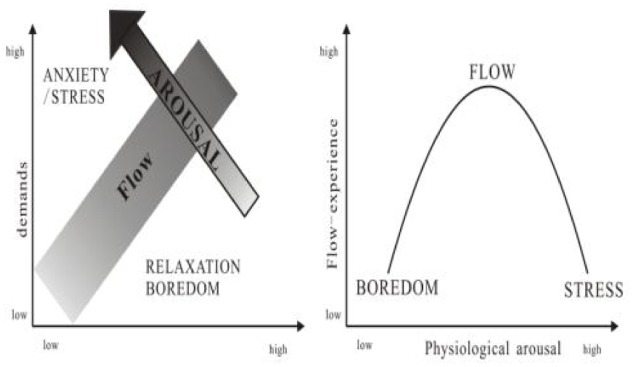
Relationship between physiological arousal and flow (Csikszentmihalyi and LeFevre, 1989).

A challenging task is a crucial precursor in the PAT model (Finneran and Zhang, 2003; Mauri et al., 2011). However, a challenging task can both facilitate and hinder flow (Fullagar et al., 2013). The challenge–skill balance model (**Figure 2**) posits that if the demands of a situation or task exceed a person’s skills and coping resources, that person experiences anxiety (Csikszentmihalyi, 1975), which has also been referred to as “stress” (Lazarus et al., 1980); in the present paper, the term “anxiety” is employed. However, if a task is insufficiently challenging, the person experiences boredom or relaxation. Flow occurs when skills and demands are in balance (Csikszentmihalyi, 1975; Lazarus and Folkman, 1984). According to flow theory, boredom and anxiety are the antithesis of flow (Csikszentmihalyi, 1975; Fullagar et al., 2013). The states of low and excessive arousal generated by boredom and anxiety were found to be associated with disintegrated attention rather than focused attention, which is characteristic of flow (Izard, 1977). This indicates that optimal physiological arousal should facilitate flow, whereas low or excessive physiological arousal should hinder it (**Figure 2**; Bressler and Bodzin, 2013; Peifer et al., 2014; Harmat et al., 2015).

Different personal characteristics can lead to substantially different flow experiences for the same activity (Csikszentmihalyi and Csikszentmihalyi, 1988; Asakawa, 2009). Such differences include not only personal skills but also a person’s underlying life attitude and personality traits (Csikszentmihalyi and LeFevre, 1989; McCrae and Costa, 1990). Shyness is a personality trait that is characterized by anxiety toward meeting people and derives from a fear of being evaluated and rejected. Shy people tend to have less self-confidence, low self-esteem, and excessive self-focused attention and self-consciousness (Pilkonis, 1977). By contrast, flow experiences are characterized by undivided attention to a limited stimulus field and an absence of self-referential thoughts (Csikszentmihalyi and LeFevre, 1989). Accordingly, when shy people interact with a CME, they are likely to assess themselves regularly to ensure that they have performed adequately. Consequently, they cannot concentrate on a task and are likely to focus on their physical environment instead. In addition, the balance of perceived challenges and skills has a central role in facilitating optimal experience, enabling a person to meet an increase in demand with a sustained level of efficacy but without an increase in mental effort (Fullagar et al., 2013). In a CME, if the perceived difficulty of a challenge is increased, the participant’s focus and information-processing speed should also increase (e.g., when developing a search strategy or using hardware and software). By contrast, excessively self-focused attention may cause shy participants to ignore information crucial to the completion of a task, to require additional effort to maintain their challenge–skill balance, and they may thus experience higher levels of anxiety. Therefore, the anxiety caused by the difficulty of the perceived challenge causes them to feel disconnected from their environment and incapable of experiencing flow. Thus, the interaction of shyness and the consequent difficulty of the task may constitute a predictor of flow experience. However, this has yet to be tested empirically. Hence, the following research hypotheses were proposed:

Hypothesis 1. Flow experience and task difficulty exhibit an inverted U-shaped relationship (self-reported).
Hypothesis 2. Shyness (shy and non-shy) is a negative predictor flow experience (self-reported).
Hypothesis 3. The interaction between shyness and task difficulty influences flow experience (self-reported).

### Flow and Physiological Signals

Previous studies have failed to ascertain the features of the physiological responses of the flow state and have only used interviews and questionnaires, which are inherently retrospective (Tezuka et al., 2014). Crucially, flow occurs during an activity in which a person is fully immersed and self-referential thoughts are completely inhibited. When participants are asked to recount their experiences in interviews and questionnaires, they have already left the flow state and have begun self-reflection. Thus, the self-report method is subjective and performed after the activity. A solution to this problem is to adopt physiological flow indicators that are objective and can be measured during the activity without interrupting the participant (Engeser, 2012).

Researchers are becoming increasingly more focused on psychophysiological investigations of flow, with the objective of identifying physiological indicators for the development of a multidimensional psychophysiological evaluation system. Many studies have suggested that the components of flow are mediated by positive valence and high arousal (e.g., Nacke and Lindley, 2009; De Manzano et al., 2010; Mauri et al., 2011; Bressler and Bodzin, 2013; Peifer et al., 2014; Harmat et al., 2015). Furthermore, the flow state is experienced by people when they are deeply and actively involved in a task, in particular game playing, which involves performing at peak ability and applying high levels of concentration, thus indicating a state of heightened arousal. Flow’s link to affect and arousal necessarily entails the flow experience having valenced content. Several components of the flow experience have been found to be dependent on positive valence and high arousal. Arousal stimuli can modulate attentional processes (i.e., concentration) (Brosch et al., 2008; Jefferies et al., 2008; Sheth and Pham, 2008); positive valence generally reduces self-awareness (Roy et al., 2008); and sense of time is altered such that positively valenced, highly arousing stimuli are perceived as being of shorter duration and are reproduced at a faster tempo than negative, less-arousing stimuli (Droit-Volet and Meck, 2007; Noulhiane et al., 2007). Thus, positive valence and high arousal are two potential physiological indicators of flow experience.

Cardiac and respiratory activity, and average changes of skin conductance (SC), are significant physiological predictors of flow experience (e.g., Uijtdehaage and Thayer, 2000; Nacke and Lindley, 2009; Mauri et al., 2011; Bressler and Bodzin, 2013; Peifer et al., 2014; Harmat et al., 2015). Typically, respiration (RSP) is rapid and shallow and has an increased minute volume (De Manzano et al., 2010), cardiovascular measures show an increase in heart rate (HR) (Uijtdehaage and Thayer, 2000; De Manzano et al., 2010), and SC (i.e., the ability of the skin to conduct an electrical current) is increased (Nacke and Lindley, 2009; Bruya, 2010; Mauri et al., 2011). These observations have been consistently associated with positive valence and high arousal. However, it should be noted that lower HR variability (HRV) during working memory-and attention-demanding tasks can be an indicator of lower mental effort, which relates to the effortless attention experienced during the flow state (Bruya, 2010; De Manzano et al., 2010; Keller et al., 2011).

We investigated the relationship between physiological signals and flow experience by examining the activation of the sympathetic and parasympathetic nervous systems. The sympathetic nervous system is the counterpart of the parasympathetic nervous system and upregulates physiological arousal (Porges, 1995). Both systems can be active simultaneously and influence the arousal process independently (Berntson et al., 1991). The interaction pattern of sympathetic and parasympathetic activation can be reciprocal, positively related (coactivation or coinhibition), or uncoupled (Berntson et al., 1991). The different possibilities of sympathetic and parasympathetic interaction provide the autonomic response with higher flexibility and precision for meeting anticipated or realized environmental challenges (Berntson et al., 1991; Thayer and Lane, 2000). Previous studies have reported that increased HR is associated with sympathetic activation (Porges, 1995) and is usually accompanied by low HRV, demonstrating variability in the length of the cardiac interbeat interval (Porges, 1995; Lehrer, 2003). Again, an elevated respiratory rate (RR) indicates increased sympathetic activity, which is indicative of high arousal; however, increased respiratory depth (RD) is an effective indicator of a more relaxed state and may reflect increased parasympathetic activity (Wientjes, 1992; Harmat et al., 2015). In addition, increased SC is associated with high task demand and is also indicative of sympathetic activation (Lacey et al., 1963; Hugdahl, 1995; Siddle et al., 1996; Dawson et al., 2007; Bruya, 2010).

We combined flow experience with physiological measurements in a CME. In this environment, participants engage in active cognitive processing (as opposed to simply recalling or memorizing information) and focus on relevant incoming information; they also engage in developing a performance strategy and using skills to complete tasks. Although physiological measurements have been demonstrated to be an effective predictor of flow experience, consistent physiological assessment systems for studying flow in the context of CMEs are lacking. For example, Harmat et al. (2015) found that higher flow was associated with lower levels of low-frequency HRV, whereas Peifer et al. (2014) reported that lower levels of low-frequency HRV were associated with lower levels of flow. Therefore, further research is required to clarify the relationship between flow experience and physiological signals. Moreover, CMEs are a crucial area requiring further exploration because of their excellent potential for promoting Web-based learning (Mauri et al., 2011), communication (Zhou, 2013), and game playing (Rheinberg et al., 2003; Keller and Bless, 2008; Moller et al., 2010; Keller et al., 2011; Bressler and Bodzin, 2013; Peifer et al., 2014; Harmat et al., 2015). Flow studies have typically centered on the balance of challenges and skills (De Manzano et al., 2010; Bressler and Bodzin, 2013; Harmat et al., 2015). However, to elucidate the causes of flow experience, we focused on the interaction of shyness and difficulty. Hence, the following hypotheses were proposed:

Hypothesis 4. Flow experience is associated with (i) increased HR, (ii) decreased HRV, (iii) increased RR, (iv) increased RD, and (v) increased SC.
Hypothesis 5. Shyness (shy and non-shy) is a negative predictor of flow experience (physiological signals).
Hypothesis 6. The interaction between shyness and task difficulty influences flow experience (physiological signals).

### The Present Study

A CME entailing a complex and demanding computer game (Blocmania 3D) that has varying difficulty levels (difficult, medium, and easy) was used as an experimental task for inducing flow according to the PAT model. During game playing, the physiological signals of the participants (shy and non-shy) were recorded continuously. The research had two aims: (1) to ascertain which physiological signals contribute to flow experience in CMEs, and (2) to ascertain whether flow experience is influenced by its precursors.

## Materials and Methods

### Method

The present study was conducted in accordance with the 1964 Helsinki declaration and its later amendments or comparable ethical standards, with the approval of the Human Research Ethics Committee of Shandong Normal University.

### Participants

A total of 350 undergraduates in China were assessed according to the College Students Shyness Scale (Wang et al., 2009). The 20 highest-scoring undergraduates were designated “shy” and the 20 lowest-scoring students were designated “non-shy.” All 40 were recruited to participate in the experiment voluntarily. The tested students were aged 17–24 years (*M* = 19.06 ± 2.14, 27 females). They were all healthy and were requested not to smoke or drink alcohol for 48 h before the experiment because this would affect the central autonomic nervous system. Written informed consent was obtained from all adult participants and from the legal guardians of all non-adult participants. All participants were informed that they had the right to withdraw from the study at any time. After completing the experiment, they were given three small gifts.

### Experimental Materials and Task

For the experiment, a popular computer game, Blocmania 3D, was employed. The original source code was retrieved from http://www.verycd.com. The game is essentially a 3D version of Tetris, the idea for which was created by Golomb (1994) and entails geometrical objects called “tetrominoes,” consisting of four squares that are joined edge-to-edge in different configurations, falling from the top of the computer screen vertically, one at a time. While a tetromino falls, the player can rotate it and move it sideways by using the up and down arrow keys on a computer keyboard. The task is to fit the pieces together and create complete horizontal rows of squares, which disappear when completed and earn the player points. In the present implementation of the game, the speed at which the pieces fall can be varied by 2 s and corresponds to the difficulty levels. We controlled the differences in difficulty among the experimental conditions by creating an additional fixed-effect task demand as a continuous variable; thus, according to the starting speed, each condition was coded as 3, 2, or 1 (difficult, medium, and easy, respectively).

To ensure variation in psychological flow during the experiment, the participants played three trials of Blocmania 3D, each with a different difficulty level. A preliminary experiment was conducted to ensure the difficulty of each condition matched the intended level of difficulty. Moreover, to provide a criterion for evaluating gameplay difficulty, the participants completed one questionnaire item, which was assessed using a 9-point Likert scale ranging from 1 (*extremely easy*) to 9 (*extremely difficult*). Analysis of the differences in perceived difficulty among the experimental conditions revealed significant differences [*F*(2,36) = 5.41, *p* < 0.01, $\eta_{p}^{2}$ = 0.23]. Pairwise comparisons indicated that the difficulty of the medium sessions (*M* = 5.41, SEM = 0.82) was significantly higher than that of the easy sessions [*M* = 3.12, SEM = 0.17; *t*(18) = 4.38, *p* < 0.01] and lower than that of the difficult sessions [*M* = 8.79, SEM = 0.53; *t*(18) = 6.31, *p* < 0.01].

### Design

In accordance with Finneran and Zhang’s (2003) elucidation of flow theory in their PAT model, the independent variables employed in the present study were the precursors of flow experience, namely shyness and task difficulty, and the dependent variables were the physiological signals and self-reports of flow experience. In this study, a two-factor mixed-design was employed. Specifically, the experiment involved three levels of within-subject task difficulty × two levels of between-subject shyness (shy and non-shy).

### Physiological Measures and Instruments

A Biopac MP 150 System (Biopac Systems Inc., Santa Barbara, CA, United States) using AcqKnowledge 4.3 was applied to continuously record the participants’ physiological signals while they were in a flow state. A sampling rate of 1000 Hz was used for all channels. For all measurements, we used the BioNomadix system as the standard filter setting.

#### Cardiovascular Activity

Cardiovascular activity was recorded by applying bipolar EL 504 Cloth Base Electrodes to the left and right sides of the participants’ chests with a 3 × 30-cm Electro Lead (BN-EL30-LEAD3) connected to a Biopac BioNomadix RSP and electrocardiogram (ECG) amplifier. The participants’ skin was cleaned using a low-alcohol detergent to minimize impedance. Generally, the quality of the recorded data was high and minimal interference was caused by movement. The recorded ECG data were imported into AcqKnowledge 4.3 to calculate the HR and associated HRV. HR was estimated from the cardiac interbeat intervals. An algorithm based on wavelet transforms was used to obtain the cardiac interbeat intervals from the ECG data. HRV refers to changes in the cardiac interbeat intervals over time. It is induced by autonomic activity, and the power spectral components of cardiac interbeat intervals provide a measurement of sympathetic and parasympathetic activity (Porges, 1995; Lehrer, 2003).

#### Respiration Activity

Thoracic RSP was measured using a piezoelectric respiratory belt transducer (MLT1133, AD Instruments) with an output range of 20–400 mV and a sensitivity of 4.5 ± 1 mV/mm. The belt was attached around the chest below the nipple line (or below the breast for women). Furthermore, RSP activity mainly entails RD and the interval of thoracic RR. We measured RR in beats per minute (bpm) and RD from peak to peak.

#### Skin Conductance

Skin conductance was recorded using a BioNomadix Electrodermal Activity Transducer connected to a BioNomadix two-channel electrodermal activity amplifier. SC was recorded using two 30-mm unpolarizable round electrodes (Clark Electromedical Instruments) placed on the middle phalanx of the index and third digits of the non-dominant hand and secured with adhesive tape. Resistance was measured with a 15-μA direct current. Additionally, the participants’ skin was cleaned using a low-alcohol detergent to minimize impedance (Rachow et al., 2011).

### Flow Experience Measures

Flow experience was measured using the Flow Short Scale to evaluate the physiological signal measures. The scale has been shown to be a reliable measuring instrument (Engeser and Rheinberg, 2008; Engeser, 2012). The scale comprises 10 items (e.g., “I do not notice time passing” and “I have no difficulty concentrating”) and a 7-point Likert scale ranging from 1 (*strongly disagree*) to 7 (*strongly agree*). The reliability of the scale is high (Cronbach’s α = 0.90). Additionally, the results of a confirmatory factor analysis, which was performed using Mplus 7.0, were as follows: χ^2^/*df* = 2.46, *p* < 0.001; comparative fit index (CFI) = 0.97; non-normed fit index (NNFI) = 0.96; root mean square error of approximation (RMSEA) = 0.04; standardized root mean square residual (SRMR) = 0.05; and all factor loadings for indicators measuring the same construct were statistically significant. The participants were asked to complete the scale immediately after completing each level.

### Experimental Procedure

The participants were asked not to consume alcoholic beverages or cigarettes for 48 h before the experiment. All experiments were conducted between 2 and 6 PM to minimize the effect of circadian variations on the physiological signals. The participants were tested individually. After arriving at the laboratory, they were briefed on the experimental procedures and fitted with the electrodes. During the experiment, a 4-min baseline measure was recorded for each participant. The participants were exposed to each experimental condition for 6 min. The Flow Short Scale was completed after each exposure. The Latin square experimental design was adopted to counterbalance the sequence effects, and the entire experimental session was completed in approximately 45 min. Additionally, to provide a criterion for evaluating whether the each participants was fond of the game, the participants completed one item after gameplay, which was assessed using a 7-point Likert scale ranging from 1 (*extremely unenjoyable*) to 7 (*extremely enjoyable*). An independent samples *t* test indicated no difference in self-reported scores between the shy and non-shy participants [*t*(38) = 0.24, *p* = 0.82].

### Statistical Analysis

All data were stored on a disk after each trial and then analyzed offline. All participants performed the cover task accurately (i.e., all of them demonstrated competence at playing Tetris); therefore, none of them were excluded from the analysis. To investigate the hypotheses, the results were analyzed using following strategies:

(a) Hypothesis 1 was a precondition for Hypothesis 3–6, in that it could elucidate how different sessions produce different experiences. Furthermore, it could clarify how the experience of a certain session contributes to flow (self-reported).
(b) Through pairwise comparisons, Hypothesis 4 could clarify which physiological signals contribute to flow (Aim 1).
(c) Hypothesis 5 could confirm whether differences in physiological activity between shy and non-shy people contribute to flow. Additionally, self-reported flow experience (Hypothesis 2) was also tested, which could further verify the effects of physiological activity on flow.
(d) Hypothesis 6 could clarify whether there the shyness–difficulty interaction effect on physiological activity contributes to flow. Additionally, self-reported flow experience (Hypothesis 3) was also tested, which could further verify the influence of physiological flow (Aim 2).

Thus, repeated measures analysis of variance (ANOVA) was applied to determine the effects of shyness and difficulty on self-reported flow and physiological flow. SPSS version 19.0 was used for the analysis. Moreover, mixed ANOVA for repeated measures was applied to determine the interaction effects between shyness and difficulty on self-reported flow and physiological flow.

## Results

**Table 1** provides the descriptive statistics for both the psychological and physiological variables.

**Table 1:** Descriptive statistics for self-reported flow experience and physiological variables for the three experimental conditions.

MEASURE	CONDITIONS					
	
	Easy		Medium		Difficult	
			
	Shy	Non-shy	Shy	Non-shy	Shy	Non-shy
Flow experience	48.57 (6.96)	49.59 (7.32)	52.38 (7.99)	56.78 (8.41)	50.05 (9.68)	52.38 (9.46)
Physiological signals						
HR	75.91 (9.68)	75.40 (9.87)	78.78 (10.53)	77.53 (10.55)	79.29 (9.42)	77.84 (9.79)
HRV	0.08 (0.05)	0.08 (0.04)	0.06 (0.05)	0.06 (0.05)	0.05 (0.02)	0.05 (0.03)
RR	19.84 (4.37)	19.74 (4.26)	21.22 (5.38)	21.40 (5.48)	22.29 (5.06)	22.39 (5.24)
RD	0.27 (0.17)	0.29 (0.19)	0.34 (0.18)	0.34 (0.17)	0.26 (0.13)	0.27 (0.15)
SC	5.56 (3.65)	5.79 (3.98)	8.32 (5.42)	8.65 (5.52)	9.38 (6.12)	9.24 (6.22)


*Values are means with standard deviations for the shy (*n* = 20) and non-shy (*n* = 20) participants. n, group size; HR, heart rate; HRV, heart rate variability; RR, thoracic respiratory rate; RD, thoracic respiratory depth; SC, skin conductance.*

The repeated measures ANOVA results showed that flow experience (self-reported) differed significantly among the three task difficulty levels [*F*(2,36) = 5.87, *p* < 0.01, $\eta_{p}^{2}$ = 0.25]. Pairwise comparisons indicated that the frequency of flow experience was significantly higher for the medium sessions (*M* = 54.53, SEM = 1.69) than for the easy sessions [*M* = 49.05, SEM = 1.71; *t*(18) = 4.56, *p* < 0.001] and difficult sessions [*M* = 49.78, SEM = 1.72; *t*(18) = 4.12, *p* < 0.001]. Thus, Hypothesis 1 was confirmed, indicating that experiencing a medium level of task difficulty contributes to flow. These results show that physiological activity induced by medium-difficulty sessions contributes to flow.

To investigate Hypothesis 4, we tested the relationships between cardiovascular activity, RR, SC, and flow experience for the three levels of task difficulty. The repeated measures ANOVA revealed significant differences in HR [*F*(2,36) = 10.59, *p* < 0.01, $\eta_{p}^{2}$ = 0.31], HRV [*F*(2,36) = 5.33, *p* < 0.05, $\eta_{p}^{2}$ = 0.23], RR [*F*(2,36) = 10.64, *p* < 0.01, $\eta_{p}^{2}$ = 0.37], RD [*F*(2,36) = 4.62, *p* < 0.05, $\eta_{p}^{2}$ = 0.21], and SC [*F*(2,36) = 13.62, *p* < 0.01, $\eta_{p}^{2}$ = 0.46] among the three levels of task difficulty. Pairwise comparisons indicated that the HR of medium sessions (*M* = 78.52, SEM = 2.21) was significantly higher than that of the easy sessions [*M* = 76.26, SEM = 2.24, *t*(18) = 3.26, *p* < 0.01] and significantly lower than that of the difficult sessions [*M* = 79.27, SEM = 2.42; *t*(18) = -2.74, *p* < 0.05]. The HRV of the medium sessions (*M* = 0.06, SEM = 0.01) was significantly lower than that of the easy sessions [*M* = 0.10, SEM = 0.17; *t*(18) = -2.38, *p* < 0.05] and higher than that of the difficult sessions [*M* = 0.03, SEM = 0.01; *t*(18) = 2.31, *p* < 0.05]. The RR of the medium sessions (*M* = 21.31, SEM = 1.24) was significantly faster than that of the easy sessions [*M* = 19.79, SEM = 1.00; *t*(18) = 3.26, *p* < 0.01], but no difference in RR was observed between the medium and difficult sessions [*M* = 22.34, SEM = 1.16; *t*(18) = 1.94 *p* = 0.64]. RD during the medium sessions (*M* = 0.34, SEM = 0.04) was significantly deeper than that during the easy sessions [*M* = 0.28, SEM = 0.04; *t*(18) = 2.15, *p* < 0.05] and difficult sessions [*M* = 0.26, SEM = 0.03; *t*(18) = 3.12, *p* < 0.01]. The CS during the medium sessions (*M* = 8.25, SEM = 1.33) was significantly higher than that during the easy sessions [*M* = 5.91, SEM = 0.95; *t*(18) = 3.69, *p* < 0.01] and lower than that during the difficult sessions [*M* = 9.38, SEM = 1.50; *t*(18) = -2.64, *p* < 0.05]. Thus, Hypotheses 4(iii) and 4(iv) were confirmed; whereas, flow experience was associated with moderate HR, HRV, and SC.

To investigate Hypothesis 2 and 5, we tested the effect of shyness (shy and non-shy) on physiological flow experience and self-reported flow experience. Repeated measures ANOVA showed no significant effects of shyness in predicting both physiological flow experience and self-reported flow experience.

**FIGURE 3:**
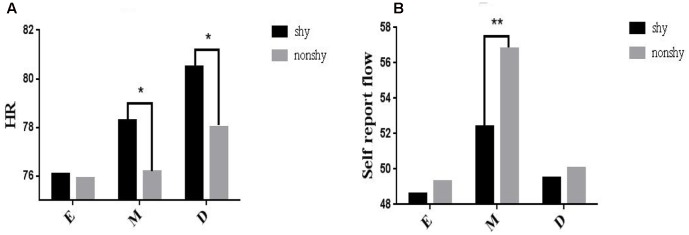
Interaction of shyness and difficulty in predicting physiological flow (HR; **A**) and the self-reported flow **(B)**. E, easy; M, medium; D, difficult. ^∗^Correlation is significant at 0.05 level; ^∗∗^correlation is significant at 0.01 level.

To investigate Hypothesis 6, a series of mixed ANOVAs was conducted to examine the interactions between shyness and difficulty in predicting flow experience (physiological activity). Crucially, we observed a significant two-way interaction between shyness and difficulty in predicting HR [*F*(2,34) = 4.56, *p* < 0.05, $\eta_{p}^{2}$ = 0.23]. Simple main effect analysis revealed that the shy participants had a higher HR than the non-shy participants under the medium [*M* = 78.78, SEM = 3.14 vs. *M* = 77.53, SEM = 3.10; *F*(2,17) = 4.31, *p* < 0.01] and difficult conditions [*M* = 79.29, SEM = 3.12 vs. *M* = 77.84, SEM = 3.23; *F*(2,17) = 3.31, *p* < 0.05]; for the easy condition, the effect was non-significant [*M* = 75.40, SEM = 3.58 vs. *M* = 75.91, SEM = 3.14; *F*(2,17) = -1.31, *p* = 0.87] (**Figure 3A**). Additionally, we also found a significant two-way interaction between shyness and difficulty [*F*(2,34) = 5.12, *p* < 0.001, $\eta_{p}^{2}$ = 0.28] in predicting the self-reported flow experience (Hypothesis 3). Simple main effect analysis revealed that the self-reported flow experience of the non-shy participants was higher that of the shy participants under the medium condition [*M* = 56.78, SEM = 2.14 vs. *M* = 52.38, SEM = 2.58; *F*(2,17) = 4.31, *p* < 0.01]; the effects were non-significant under the easy condition [*M* = 49.59, SEM = 2.78 vs. *M* = 48.57, SEM = 2.63; *F*(2,17) = 1.43, *p* = 0.69] and difficult condition [*M* = 50.05, SEM = 3.12 vs. *M* = 52.38, SEM = 2.74; *F*(2,17) = 1.51, *p* = 0.43] (**Figure 3B**).

## Discussion

### Flow and Physiological Signals

On the basis of the TAP model, we investigated the physiological activity of participants in a flow state while they played the game Blocmania 3D. Repeated measures ANOVA results show that self-reported data support the experimental manipulation (i.e., the speed of falling tetrominoes) under the different experimental conditions, and pairwise comparisons demonstrated that the optimal flow experience occurred during the medium session. Furthermore, pairwise comparisons of the physiological measurements demonstrate that moderate HR, moderate HRV, increased RR, moderate RD, and moderate SC were evident when the participants were in the optimal flow state.

During game playing, participants experience feelings of enjoyment that indicate a state of heightened arousal and positive affect (Harmat et al., 2015). This may be because more key nutrients are consequently required; therefore, HR increases to meet the metabolic demand and increased cardiac output. Furthermore, a rapid RR increases the efficiency of oxygenation (oxygen in the lungs has maximal access to the heart). Thus, flow experience may be associated with increased metabolism related to sympathetic nervous activity. However, the higher RD during high flow indicates a more relaxed state and elevated parasympathetic activity; thus, a non-reciprocal increase of activity in both branches of the autonomic nervous system may indicate increased parasympathetic modulation of sympathetic activity. This combination of cardiorespiratory patterns agrees with the findings of previous studies (De Manzano et al., 2010; Mauri et al., 2011; Peifer et al., 2014; Harmat et al., 2015). However, this does not explain why the HRV changes did not occur in the expected direction. HRV may be used as an indicator for mental effort in flow (Bruya, 2010; De Manzano et al., 2010; Keller et al., 2011). Relatively high HRV values (compared with HRV levels in a relaxed and stressful state) indicate that increasing task difficulty could result in greater mental effort and that flow experience is a state of moderate mental effort.

Moderate sympathetic activity during flow was indicated by moderate SC values relative to that under a relaxed or stressful state; however, comparably significant results were not observed for HR or RR. SC appears to be a physiologically sensitive indicator of sympathetic activity. Thus, SC patterns provide a specific signature of the state of flow, which is associated with moderate sympathetic activity. Additionally, because mental effort is positively associated with sympathetic activity (De Manzano et al., 2010; Harmat et al., 2015), the moderate SC values, which are related to moderate sympathetic activity, also indicate that flow experience is a state of moderate mental effort.

### Mediation of Flow Precursors

The physiological assessment system for studying flow experience in the context of CMEs exhibited favorable psychometric properties with respect to our first aim. This assessment system was used to test the second aim, namely whether flow experience is moderated by its precursors. Mixed ANOVA results demonstrate that the interaction of shyness and task difficulty can predict HR, and the simple main effect analysis results show that the HR of the shy participants was higher than that of the non-shy participants in the medium and difficult sessions. However, flow experience was related to moderate HR, indicating that the shy participants may have had a lower flow experience than the non-shy participants in the medium session. Additionally, the mixed ANOVA results for self-reported flow also show that the flow experience of the shy participants was lower than that of the non-shy participants in the medium session, and this result further verified the physiological flow (moderate HR). Thus, Aim 2 was verified, and the PAT model was found to be successful in measuring flow in CME.

The challenge–skill balance indicates that if a person’s skills are too great for a given task, that person will experience boredom; by contrasting, if a task is too challenging, the person will experience anxiety (Csikszentmihalyi, 1975). In Blocmania 3D, the participants had different experiences as the difficulty level increased. At the easy level, the speed of the falling pieces was slower and the participants could easily create complete horizontal rows of squares; consequently, they experienced boredom. By contrast, high levels of difficulty lead to anxiety. However, at the medium level, which was the optimal level of challenge, completing the tasks elicited feelings of competence (Deci and Ryan, 2000). Perceived competence tends to occur naturally in players when they are in a flow state.

As the speed of the falling pieces increased, we expected that the participants would be more focused on the task and process information quicker; this required them to use the keyboard to rotate squares and develop a strategy for creating complete horizontal rows of squares. However, too much self-focused attention may have caused the shy participants to ignore critical information required for developing such a strategy, and they may have required additional mental effort to maintain the challenge–skill balance. Furthermore, significant differences were observed between the medium and difficult levels but not for the easy level. This could be because at this level, minimal mental effort was required and thus both the shy and non-shy participants experienced boredom rather than flow. However, for the medium and difficult levels, the shy participants applied more mental effort; however, the flow experience is associated with moderate mental effort. Accordingly, the flow experience for shy people might be low at the medium and difficult levels.

In addition, shyness has been found to negatively influence the frequency of flow experience (Hirao et al., 2012). This is because flow is experienced when a high-level challenge is balanced with high-level skills and intrinsic rewards are generated, and it is negatively associated with subjective anxiety. However, shy people tend to have low confidence. Therefore, the authors of that study contended that it is possible that a lack of confidence can prevent shy people from reaching the flow state when faced with a highly challenging task. Accordingly, in the case of shy people, to reach the flow state, their ability to complete a task must be improved or the level of a challenge must be reduced.

Physiologically, HR was successful in measuring the mediation of flow precursors in CME, which is associated with sympathetic activity. During gameplay, the HR of the shy participants was higher than that of the non-shy participants under the medium and difficult conditions. This suggests that the shy participants required more oxygen to meet their metabolic demand and that increased mental effort consumed more oxygen and nutrients. By contrast, no significant difference was observed for RR, which is another significant indicator of sympathetic activity. This discrepancy could be attributable to RR being self-regulated subjectively and shy players possibly adjust their behaviors regularly to maintain a moderate RR, whereas HR is impossible to self-regulate and is thus objective. Therefore, flow experience is mediated by its precursors in CMEs (Guo and Poole, 2009; Mauri et al., 2011).

### Limitations and Future Directions

First, a conceivable limitation of the present study was that the level of difficulty was not adapted to the skill level of each participant individually. Despite the results of self-reporting indicating a significant difference among the three experimental difficulties, matching the speed to each participant’s skill is difficult. Future studies should match the challenge to each participant’s skill for participants to experience the optimal flow experience. Second, this study examined only the effect of person (P) and task (T) on flow experience in a CME context; future studies should consider investigating the influence of all factors (person, artifact, and task) of the PAT model on flow experience. In addition, the contributions of different physiological indicators require exploration, because different indicators may have different weights in predicting flow experience, such as event-related potentials and electroencephalograph signals, which could reveal brain activity.

## Conclusion

First, the results show that moderate HR, moderate HRV, increased RR, moderate RD, and moderate SC are related to flow experience. Second, flow experience during gameplay is the result of increased parasympathetic modulation of sympathetic activity. Third, flow experience is influenced by its precursors, and HR is an effective indicator for measuring the interaction of flow precursors in a CME context.

## Author Contributions

YT contributed to writing, data analysis, and polishing the manuscript. PH conducted experiments and data analysis. YB, PW, FG, and YC also contributed in polishing the manuscript.

## Conflict of Interest Statement

The authors declare that the research was conducted in the absence of any commercial or financial relationships that could be construed as a potential conflict of interest.
